# Supplementary material for: A Realistic In Silico Brain Phantom for Quantifying Susceptibility Anisotropy‐Induced Error in Susceptibility Separation
Source: Magn Reson Med. 2026 Jul 9;96(5):2412–25. doi: 10.1002/mrm.70468 (PMC13527262; doi:10.1002/mrm.70468)
Supplement: Supplementary file 1 [file MRM-96-2412-s001.docx]

Supplementary material

1) **Values of** *C* and<https://www.codecogs.com/eqnedit.php?latex=%5Cbeta#0>

The<https://www.codecogs.com/eqnedit.php?latex=T_1#0> model parameters (C and β) were determined using a least squares fitting approach based on the values reported by Rooney et al. [39] for multiple brain regions. The results, presented in Table 1, show that<https://www.codecogs.com/eqnedit.php?latex=T_1#0> values generally increase with B0, consistent with known tissue relaxation behavior. However, the CSF appears to be an exception, with its<https://www.codecogs.com/eqnedit.php?latex=T_1#0> remaining approximately constant across field strengths, as indicated by a near-zero<https://www.codecogs.com/eqnedit.php?latex=%5Cbeta#0> value.

| **ROIs** | **T_1_ model** |
| --- | --- |
| Caudate nucleus | T_1_=0.954(B_0_)^0.325^ |
| Globus pallidus | T_1_=0.664(B_0_)^0.367^ |
| Putamen | T_1_=0.855(B_0_)^0.352^ |
| Red nucleus | T_1_=0.459(B_0_)^0.508^ |
| Dentate nucleus | T_1_=0.459(B_0_)^0.508^ |
| Substantia nigra | T_1_=0.459(B_0_)^0.508^ |
| Thalamus | T_1_=0.817(B_0_)^0.357^ |
| White matter | T_1_=0.583(B_0_)^0.376^ |
| Grey matter | T_1_=0.857(B_0_)^0.376^ |
| CSF | T_1_=4.322(B_0_)^-0.006^ |

*Table S1: T_1_ model for QSM validation phantom ROIs obtained by a least square fit of the model proposed by Rooney et al.,* [*[39]*](https://paperpile.com/c/xIG4Dt/OzHL)*.*

2) **3 T vs 7 T Bland Altman**

Figure S2 shows Bland-Altman analyses of the measured 3 T and 7 T<https://www.codecogs.com/eqnedit.php?latex=%5Cchi%5E-#0> in WM sub-ROIs with susceptibility anisotropy effects. The plots indicate a bias of 0.0034, -0.0024, 0.0057, and -0.0109 ppm for<https://www.codecogs.com/eqnedit.php?latex=%5Cchi#0>-separation,<https://www.codecogs.com/eqnedit.php?latex=R_2%5E*#0>-QSM, APART-QSM, and DECOMPOSE-QSM, respectively.

Overall, the Bland-Altman analysis shows a weak systematic difference as most points are within the lines of agreement, with a more pronounced bias in DECOMPOSE-QSM.

*Figure S1: Bland–Altman plot comparing the measured*<https://www.codecogs.com/eqnedit.php?latex=%5Cchi%5E-#0>*values using four susceptibility separation techniques from 3 T and 7 T simulated data with susceptibility. Each data point represents the averaged susceptibility value within a WM sub-ROI.*

3)<https://www.codecogs.com/eqnedit.php?latex=%5Cchi%5E%2B#0> **simulated vs measured**

Figure S2 shows kernel density estimate (KDE) plots of the WM sub-ROI-averaged MSPE values for<https://www.codecogs.com/eqnedit.php?latex=%5Cchi%5E%2B#0> measured from noiseless 3 T data simulated with and without susceptibility anisotropy effects. For algorithms that use<http://www.texrendr.com/?eqn=R_2%5E'#0> (<https://www.codecogs.com/eqnedit.php?latex=%5Cchi#0>-separation and APART-QSM), the MSPE is higher when susceptibility anisotropy is included. However, for algorithms that use<https://www.codecogs.com/eqnedit.php?latex=R_2%5E*#0> (DECOMPOSE-QSM and<https://www.codecogs.com/eqnedit.php?latex=R_2%5E*#0>-QSM), the MSPE is lower when susceptibility anisotropy is included. MSPE values for<https://www.codecogs.com/eqnedit.php?latex=%5Cchi%5E%2B#0> are considerably large, highlighting the limitations of these methods to the small susceptibility values encountered in WM in<https://www.codecogs.com/eqnedit.php?latex=%5Cchi%5E%2B#0>.

*Figure S2: KDE plots of the WM sub-ROI-averaged MSPE of*<https://www.codecogs.com/eqnedit.php?latex=%5Cchi%5E%2B#0> *measured from noiseless 3 T data simulated* *with (blue) and without (orange) susceptibility anisotropy using four susceptibility separation techniques:*<https://www.codecogs.com/eqnedit.php?latex=%5Cchi#0>*-separation,*<https://www.codecogs.com/eqnedit.php?latex=R_2%5E*#0>*-QSM, APART-QSM, and DECOMPOSE-QSM.*

Figure S3 shows KDE plots of ROI-averaged<https://www.codecogs.com/eqnedit.php?latex=%5Cchi%5E%2B#0> MSPE measured from noiseless 3 T data simulated with and without susceptibility anisotropy effects, for the deep GM and GM ROIs listed in Table 1 column 1, excluding WM.<https://www.codecogs.com/eqnedit.php?latex=%5Cchi#0>-separation achieved the lowest MSPE value, followed by APART-QSM, then<https://www.codecogs.com/eqnedit.php?latex=R_2%5E*#0>-QSM, and finally DECOMPOSE-QSM. For every algorithm tested, the distribution of MSPE with anisotropy appears to be broader and has a higher average than that of the case without anisotropy indicating that although susceptibility anisotropy was not modeled in GM it still affects the surrounding tissue.

*Figure S3: KDE plots of the*<https://www.codecogs.com/eqnedit.php?latex=%5Cchi%5E%2B#0> *MSPE measured in the deep GM and GM ROIs listed in Table 1 column 1 from noiseless 3 T data simulated* *with (blue) and without (orange) susceptibility anisotropy and fitted using four susceptibility separation techniques:*<https://www.codecogs.com/eqnedit.php?latex=%5Cchi#0>*-separation,*<https://www.codecogs.com/eqnedit.php?latex=R_2%5E*#0>*-QSM, APART-QSM, and DECOMPOSE-QSM.*
